# A rare case of peritoneal dialysis‐associated peritonitis caused by *Comamonas testosteroni*

**DOI:** 10.1111/sdi.13126

**Published:** 2022-11-04

**Authors:** Roman Kuźniewicz, Mirosław Śnit, Dariusz Szczyra

**Affiliations:** ^1^ Department of Internal Medicine, Diabetology and Nephrology, Faculty of Medical Sciences in Zabrze Medical University of Silesia Katowice Poland

## 1 INTRODUCTION

*Comamonas testosteroni* is a bacterium belonging to the genus *Comamonas*. *C. testosteroni* is a gram‐negative aerobic bacillus with high motility, and it is commonly found in soil, water reservoirs, and plants. It uses testosterone, 4‐hydroxybenzoate (4HBA), acetate, and lactate as its sole carbon sources.^1^ *C. testosteroni* has received much attention due to its property of degrading environmental pollutants and resistance to heavy metals.^1^ The fact that this bacterium exists in activated sludge or post‐mining soil contaminated with heavy metals is used to remove contamination of soil or water reservoir contaminants (in what is known as bioremediation).^2^ This bacillus has also been detected in hospital environments in the water of oxygen humidifiers and in hemodialysis solutions, but also in urine, feces, and respiratory secretions.^2^ *C. testosteroni* has low virulence, and despite its wide distribution in nature, reports of human infection are rare.

## 2 CASE REPORT

A 72‐year‐old obese Caucasian patient with generalized atherosclerosis, arterial hypertension, and end‐stage kidney disease secondary to long‐standing of Type 2 diabetes mellitus (during intensive insulin therapy), treated with continuous ambulatory peritoneal dialysis (CAPD) for 6 months, after experiencing a mild form of COVID‐19 3 months earlier, was hospitalized due to sign of first dialysis peritonitis (PD): cloudy dialysate, without other symptoms except slight abdominal tenderness on palpation. High pleocytosis of dialysate was noted: 2.73 * 10 to 3/μl, blood CRP level 67.2 mg/L with leukocytosis 15.7 * 10 to 3/μl (74.5% granulocytes), hemoglobin values: 10.8 g/dl, platelet count: 127 * 10 to 3/μl, albumin: 33.3 g/L and total protein: 56.6 g/L; glycemias within 200 mg/dl, HbA1c: 6.27%. Empirical intraperitoneal antibiotic therapy was administered (ceftazidime via intraperitoneal route), initially with unfractionated heparin; hypotensive treatment and insulin therapy were continued. Three days later bacteriological examination under aerobic conditions of dialysate yielded growth of *C. testosteroni*. Colonies were cultured on blood agar and MacConkey at 35–37°C and identified with 99% probability by colorimetric method, and antibiotic susceptibility tests were performed using a VITEK 2 Compact automated instrument (bioMérieux, France) and interpreted according to EUCAST: bacterial sensitivity to amikacin, cefoperazone/sulbactam, ceftazidime, ciprofloxacin, gentamicin, and ticarcillin and bacterial resistance to: cefuroxime, tetracycline, and trimethoprim/sulfamethoxazole. Chest computed tomography showed a right lung apical nodule stable for 4 weeks, while ultrasound and abdominal computed tomography revealed three focal liver lesions of primary hepatocellular carcinoma (HCC) or metastasis with unclear starting point. The patient consciously refused diagnostic biopsies of the lung and liver lesions under CT scan.

It was decided to continue treatment with ceftazidime via intraperitoneal route. A rapid resolution of peritonitis symptoms was achieved, on day 14 the dialysate pleocytosis was 0.025 * 10 to 3/μl, bacteriological follow‐up was negative. Over the next 6 months, except for weakness, the patient did not experience any complaints and continued CAPD.

## 3 DISCUSSION

*C. testosteroni* infection with septicemia in a 31‐year‐old woman with rheumatic heart disease was first described in 1975.^3^ Among the descriptions of *C. testosteroni* infections in the available literature, infections caused by this bacterium more often than PD involve bacteremia, endocarditis, pneumonia, connective tissue inflammation, meningitis, urinary tract infections, or abdominal organs.^4^ Cases of bacteremia occur in elderly patients,^4^ those burdened with diabetes, cancer, or alcohol or drug dependence. For example, authors from Taiwan describe bacteremia in a 54‐year‐old alcoholic with a lower limb injury and a 73‐year‐old patient with chronic hepatitis B, cirrhosis, and hepatocellular carcinoma treated with transarterial embolization; bacteremia was found after this procedure.^5^

From Iran was reported two cases of *C. testosteroni* bacteremia that developed during malignancy in a 10‐year‐old boy with brain medulloblastoma and in a 19‐year‐old woman with osteosarcoma.^6^

A lethal course of a bacteremia of *C. testosteroni* in a 64‐year‐old female patient hemodialysed with a central hemodialysis catheter was reported too.^7^ In the literature, we encountered descriptions of bloodstream infections where *C. testosteroni* was one of several bacteria causing bacteremia. On the other hand, in India, a 46‐year‐old woman with no health burden had bacteremia and urinary tract infection.^8^

In another report, PD peritonitis caused by *C. testosteroni* was described in a 29‐year‐old woman with end‐stage kidney disease secondary to hypertensive nephropathy, treated with CAPD for 10 months. In addition to chronic dialysis therapy, the predisposing factor for peritonitis with this pathogen was probably a previous laparoscopic removal of an intrauterine device (IUD) displaced into the space between the peritoneum and the abdominal wall. Symptoms of peritonitis resolved in this patient after 14 days of oral ciprofloxacin.^9^

Swiss researchers analyzing bacteriological findings of patients hospitalized over a 12‐year period in Lausanne found that *Comamonas* bacilli, including *C. testosteroni*, were isolated in 33% from the respiratory tract, 23% from the genitourinary tract, and 21% from the gastrointestinal tract, and bacteremia occurred in three patients (5%).^10^ Also, these authors first described bacteremia with a related bacillus, *Comamonas kerstersii*, in a patient with colonic diverticulosis.^10^

In our reported case, there are no data on the patient's colonic diverticulosis. On the other hand, in the pathogenesis of peritonitis in CAPD‐treated patients, translocation of enteric bacteria from the gastrointestinal tract to the peritoneal cavity via transmural migration seems to play an important role. *Comamonas* bacilli are so widespread that they can enter the patient's gastrointestinal tract and then penetrate inflammationally altered intestinal walls and causing peritonitis.^11^

Most *C. testosteroni* infections are thought to occur outside the hospital in immunocompromised patients due to malignancy, chronic liver disease, end‐stage kidney disease, and other causes.^7^

*C. testosteroni* is usually sensitive to many antibiotics including aminoglycosides, cephalosporins, fluoroquinolones, carbapenems, piperacillin–tazobactam, and trimethoprim–sulfamethoxazole. In the described cases of infection with this bacterium, a rapid response to antibiotic therapy was achieved, and this was also the case in the described case of peritonitis after intraperitoneal administration of a cephalosporin (ceftazidime). This enabled the patient to maintain the CAPD method of renal replacement therapy.

## 4 CONCLUSION

*C. testosteroni* should be kept in mind as a rare cause of bacterial peritonitis in CAPD patients. With the increasing availability of CAPD, there are an increasing number of adults with comorbidities sometimes with severe disease, which could potentially increase the susceptibility to *C. testosteroni* infections.

## CONFLICT OF INTEREST

All authors declare no conflict of interest.

## AUTHOR CONTRIBUTIONS

MS and RK researched literature and conceived the study. RK wrote the first draft of the manuscript. MS and DS reviewed and edited the manuscript and approved the final version of the manuscript.
